# miR-9-5p Exerts a Dual Role in Cervical Cancer and Targets Transcription Factor TWIST1

**DOI:** 10.3390/cells9010065

**Published:** 2019-12-26

**Authors:** Iris Babion, Annelieke Jaspers, Annina P. van Splunter, Iris A.E. van der Hoorn, Saskia M. Wilting, Renske D.M. Steenbergen

**Affiliations:** 1 Cancer Center Amsterdam, Pathology, Amsterdam UMC, Vrije Universiteit Amsterdam, 1081 HV Amsterdam, The Netherlands; 2 Department of Medical Oncology, Erasmus MC Cancer Institute, Erasmus University Medical Center, 3015 GD Rotterdam, The Netherlands

**Keywords:** cervical cancer, HPV, miR-9-5p, methylation, TWIST1

## Abstract

Squamous cell carcinoma (SCC) and adenocarcinoma (AC) represent the major cervical cancer histotypes. Both histotypes are caused by infection with high-risk HPV (hrHPV) and are associated with deregulated microRNA expression. Histotype-dependent expression has been observed for miR-9-5p, showing increased expression in SCC and low expression in AC. Here, we studied the regulation and functionality of miR-9-5p in cervical SCCs and ACs using cervical tissue samples and hrHPV-containing cell lines. Expression and methylation analysis of cervical tissues revealed that low levels of miR-9-5p in ACs are linked to methylation of its precursor genes, particularly *miR-9-*1. Stratification of tissue samples and hrHPV-containing cell lines suggested that miR-9-5p depends on both histotype and hrHPV type, with higher expression in SCCs and HPV16-positive cells. MiR-9-5p promoted cell viability and anchorage independence in cervical cancer cell lines SiHa (SCC, HPV16) and CaSki (metastasized SCC, HPV16), while it played a tumor suppressive role in HeLa (AC, HPV18). TWIST1, a transcription factor involved in epithelial-to-mesenchymal transition (EMT), was established as a novel miR-9-5p target. Our results show that miR-9-5p plays a dual role in cervical cancer in a histotype- and hrHPV type-dependent manner. MiR-9-5p mediated silencing of TWIST1 suggests two distinct mechanisms towards EMT in cervical cancer.

## 1. Introduction

Cervical cancer is caused by a persistent infection with high-risk types of the human papillomavirus (hrHPV) [1,2,3]. Squamous cell carcinoma (SCC, ca. 80%) and adenocarcinoma (AC, 10–20%) represent the most prevalent cervical cancer subtypes [4,5]. SCCs develop through a series of precancerous lesions, which arise from squamous epithelial cells present in the ectocervix, whereas columnar epithelial cells in the endocervix give rise to ACs. Moreover, most SCCs are caused by HPV16, while HPV18 is particularly linked to cervical ACs [6,7]. During cervical cancer development multiple miRNAs become deregulated, leading to aberrant gene expression and disease progression [8,9,10,11]. MiRNAs are therefore considered promising diagnostic, prognostic, and therapeutic targets.

MiR-9-5p is of particular interest as it has been described to exert a tumor suppressive function in glioblastoma multiforme and ovarian carcinoma, while it acts as oncomiR in breast, prostate, and non-small cell lung cancer [12,13,14,15,16]. This versatile function of miR-9-5p in human cancers has recently been reviewed by Nowek et al. and likely results from a variety of downstream targets in different cellular contexts [17]. Among others, cyclin D1 (CCND1), E-cadherin (CDH1), FOXO3, IL6, and NOTCH1 have been described as direct targets of miR-9-5p [17,18].

In cervical tissue samples, we found significantly increased expression of miR-9-5p in SCCs compared to healthy cervical epithelium, but not in ACs [8]. Differential expression of miR-9-5p between the two cervical cancer histotypes is also reflected in cervical scrapes of women with underlying SCC and AC [19]. The mature miR-9-5p has three independent precursors: mir-9-1, mir-9-2, and mir-9-3, which are transcribed from chromosomal regions 1q22, 5q14.3, and 15q26.1, respectively. We previously showed that upregulation of miR-9-5p in SCC is associated with a copy number gain of chromosome 1q, which occurs more frequently in cervical SCCs than ACs [8,20,21]. Zhang et al., on the other hand, reported that low expression of miR-9-5p in cervical ACs compared to healthy cervical tissue is due to hypermethylation of all three miR-9-5p precursor genes [22]. In line with these observations, miR-9-5p was shown to play an oncogenic function in HPV16-tranformed keratinocytes and HPV16-positive SiHa cells originating from a cervical SCC, while a tumor suppressive role has been described in the HPV18-positive cervical AC cell line HeLa [8,18,22]. Taken together, these studies suggest that miR-9-5p expression in cervical cancer may be cell type and/or hrHPV type-dependent and are suggestive of a dual functional role of miR-9-5p in cervical cancer.

To further elucidate the regulation and role of miR-9-5p in cervical cancer, this study aimed to comprehensively compare miR-9-5p expression, regulation, and functionality in the context of cervical cancer histotype and hrHPV type. We show that miR-9-5p expression depends on both histotype and hrHPV type and suggest two routes towards EMT in cervical cancer.

## 2. Materials and Methods

### 2.1. Cell Lines and Clinical Specimens

Cervical cancer cell lines SiHa, CaSki and HeLa, and renal epithelium cell line HEK293 were authenticated by STR testing using the Powerplex16 System (Promega, Leiden, The Netherlands) and cultured as described previously [23]. Primary human foreskin keratinocytes (HFKs) were isolated and HPV16 (FK16A and FK16B) and HPV18 (FK18A and FK18B) immortalized keratinocyte cell lines were established and cultured as described previously [24,25]. To study the effect of methylation inhibition on miR-9-5p expression, SiHa and HeLa cells were treated daily with 5 µM 5-aza-2’-deoxycytidine (5aza, Sigma-Aldrich, St. Louis, MO, USA) for four consecutive days.

For DNA methylation analysis in cervical biopsies, formalin-fixed paraffin-embedded (FFPE) biopsies of six SCC and 11 AC were used. Moreover, fresh frozen tissue specimens of 18 SCC and six AC were included. All included carcinomas were positive for HPV, as determined with the general primer GP5+/6(+)-mediated PCR-enzyme immunoassay in combination with the luminex genotyping kit HPV GP [26,27]. All samples were used in an anonymous fashion in accordance with the “Code for Proper Secondary Use of Human Tissues in the Netherlands” as formulated by the Dutch Federation of Medical Scientific Organizations (https://www.federa.org) [28].

### 2.2. RNA/DNA Isolation and Bisulfite Treatment

Total RNA was isolated from cell lines using the TRIzol reagent (Thermo Fisher Scientific, Bleiswijk, The Netherlands) according to the manufacturer’s instructions.

Genomic DNA was isolated from cell lines using the PureLink DNA mini kit (Thermo Fisher Scientific) according to the manufacturer’s protocol. DNA concentrations were determined using the NanoDrop Spectrophotometer (Thermo Fisher Scientific). DNA from FFPE and fresh frozen biopsies was isolated by standard proteinase K digestion followed by phenol-chloroform purification [29]. The Qubit^®^ dsDNA BR assay kit in combination with a Qubit^®^ 2.0 Fluorometer (both Thermo Fisher Scientific) was used to quantify DNA concentrations obtained from tissue samples. DNA was modified using the EZ DNA Methylation kit (Zymo Research, Leiden, The Netherlands) according to the manufacturer’s instructions.

### 2.3. Quantitative Methylation-Specific PCR (qMSP)

For methylation analysis of miR-9 precursors, a multiplex quantitative methylation-specific PCR (qMSP) assay was developed as previously described [30]. MSP primers were designed to specifically amplify the methylated bisulfite-converted DNA sequence of the *mir-9-1*, *mir-9-2,* and *mir-9-3* promoter regions (Table S1a). Specificity of the primer sets to methylated DNA was confirmed in modified but unmethylated DNA of HFK cells during assay development. To verify sufficient DNA quality and successful bisulfite conversion the modified sequence of β-actin (ACTB) was amplified. Multiplex qMSP was carried out using 50 ng of bisulfite-converted DNA as a template on the ABI7500 Fast Real-Time PCR System (Thermo Fisher Scientific). In vitro methylated DNA (IVD) was used as positive control, while unmodified SiHa DNA and H2O served as negative controls. Data were normalized to ACTB applying the 2^(−ΔC_t_) method [31].

### 2.4. Quantitative Reverse Transcription-PCR (qRT-PCR)

#### 2.4.1. miRNA qRT-PCR

Expression of miR-9-5p and U75 (000583, 001219; Thermo Fisher Scientific) was measured using TaqMan microRNA assays in combination with the TaqMan microRNA reverse transcription kit (Thermo Fisher Scientific). cDNA was synthesized from 10 ng total RNA template. The TaqMan Universal Master Mix II, no UNG (Thermo Fisher Scientific) was used for qPCR reactions, which were carried out on the ABI7500 fast real-time PCR system (Thermo Fisher Scientific). miRNA expression data were normalized to U75 using the 2^(−ΔC_t_) method [31].

#### 2.4.2. mRNA qRT-PCR

To quantify expression levels of EMT markers CDH1, CDH2, TWIST1, and reference gene snRNP U1A, cDNA was synthesized from 200 ng total RNA template using specific reverse primers (Table S1b). RNA samples were treated with the RQ1 DNase (Promega) prior to reverse transcription. Subsequently, qPCR reactions were performed using the 2× SYBR Green master mix or—in combination with a probe—the TaqMan Universal Master Mix II, no UNG (both Thermo Fisher Scientific) on the ABI7500 fast real-time PCR system (Thermo Fisher Scientific) according to the manufacturer’s protocol. Specificity of the SYBR Green PCR reactions was determined generating melting curves for each reaction. Data were normalized to snRNP U1A using the 2^(−ΔC_t_) method [31].

### 2.5. External Data

Expression of miR-9-5p was analyzed in microarray data of 10 healthy cervical epithelium specimens, 10 SCC and nine AC available from the Gene Expression Omnibus (GEO, http://www.ncbi.nlm.nih.gov/geo/) through series accession number GSE306568. Moreover, miR-9-5p expression was analyzed in TCGA data available from https://gdc-portal.nci.nih.gov/ and https://tcga-data.nci.nih.gov/docs/publications/cesc_2016/23.

### 2.6. Transfection with miRNA Inhibitors and Mimics

SiHa, CaSki, and HeLa cells were transiently transfected with 20 nM miRCURY LNA microRNA inhibitors for miR-9-5p, negative control A (4100536, 199066; Exiqon, Vedbaek, Denmark), or 20 nM miRIDIAN microRNA mimics for miR-9-5p and negative control #2 (C-300619-03, CN-002000-01; GE Dharmacon, Lafayette, CO, USA). Cells were transfected with Dharmafect 1 for 22 h according to the manufacturer’s instructions (GE Dharmacon, Lafayette, CO, USA). Cells were harvested for RNA 72 h after transfection.

### 2.7. Cell Viability and Anchorage-Independent Growth

Cell viability was measured using the fluorometric CellTiter-Blue assay according to the manufacturer’s protocol (Promega). Cells were seeded in triplicate in 96-well plates (2500 cells/well) 22 h after transfection. Cell viability was measured at 24 and 72 h after transfection and the average measurement after 24 h was subtracted from the measurements at 72 h. To examine anchorage-independent growth, colony formation in soft agar was analyzed as described before [25]. In short, 5000 cells were plated in a medium containing 0.35% top agarose (Seaplague agarose; Lonza Group Ltd., Basel, Switzerland) on a surface of 0.6% bottom agarose in 6 cm dishes in duplicate. After three weeks, colonies exceeding ~50 cells were counted. Each experiment was performed at least two times. Representative experiments are shown.

### 2.8. Construction of a pmirGLO Reporter Vector

The predicted TWIST1 3’UTR binding site of miR-9-5p was amplified from 100 ng template DNA by Phusion High-Fidelity PCR (New England Biolabs, Ipswich, MA, USA). 3’UTR amplicons and the pmirGLO dual-luciferase miRNA Target Expression Vector (Promega) were digested by SacI and XhoI (New England Biolabs) and ligated by a T4 DNA ligase (Roche, Woerden, The Netherlands). The predicted binding sites were mutated using the Q5 site-directed mutagenesis kit (New England Biolabs) according to the manufacturer’s instructions. Primer sequences are listed in Table S1c.

### 2.9. Luciferase Dual-Reporter Assays for miR-9-5p Target Validation

For luciferase assays, HEK293 were seeded in triplicate in 96-well plates (7500 cells/well) and co-transfected with mimics and pmiRGLO vector as described above the following day. The firefly luciferase activity was measured using the Dual-Glo luciferase assay (Promega) according to the manufacturer’s instructions 48 h after transfection. Renilla luciferase activity was determined as an internal control and used for normalization. Each experiment was carried out at least two times. Representative experiments are shown.

### 2.10. Statistical Analysis

Expression and methylation levels between sample groups were compared using the non-parametric Wilcoxon rank-sum test (two-sided). The two-sided Student’s *t*-test was used to compare in vitro experimental conditions. To study the correlation between miR-9-5p expression and TWIST1, CDH1, and CDH2, the Spearman correlation coefficient and associated *p*-value were assessed.

## 3. Results

### 3.1. Low miR-9-5p Expression in AC Is Associated with DNA Hypermethylation

**Figure 1:**
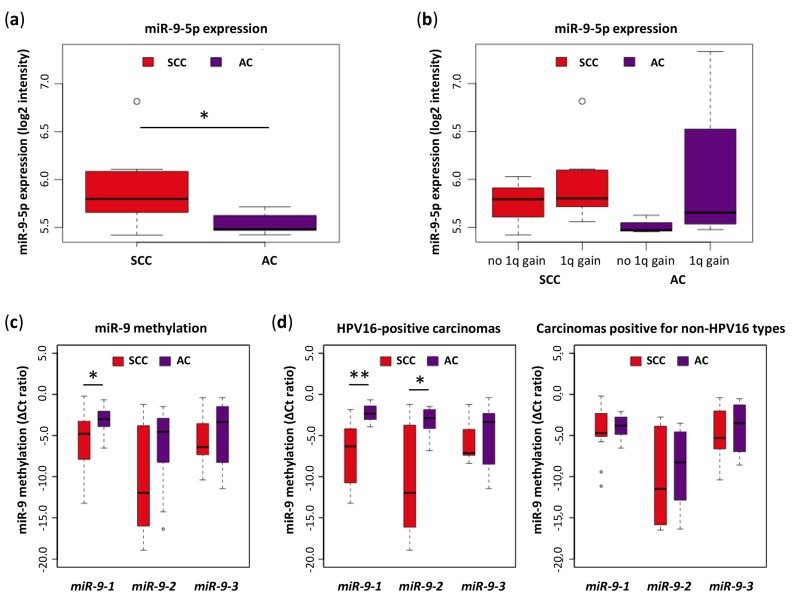
Expression of miR-9-5p and methylation of miR-9 precursors in cervical tissue samples. (**a**) Expression was determined by microarray analysis in micro-dissected SCCs (*n* = 10) and ACs (*n* = 9) [8]; (**b**) miR-9-5p expression was stratified by the presence (seven SCCs, four ACs) or absence (three SCCs, three ACs) of a 1q gain. For two ACs chromosomal data was not available; (**c**) methylation levels of miR-9-5p precursors *miR-9-1*, *miR-9-2*, and *miR-9-3* were determined by quantitative MSP in 24 SCC and 17 AC tissue samples; (**d**) methylation analysis was further stratified by hrHPV type (HPV16 or other). HPV16-positive carcinomas included 11 SCCs and eight ACs. Carcinomas positive for non-HPV16 types were comprised of 13 SCCs (of which one was positive of HPV18) and nine ACs (of which seven were positive for HPV18). SCC: Squamous cell carcinoma; AC: Adenocarcinoma. * *p* < 0.05, ** *p* < 0.005.

Our previously published miRNA microarray data show that mature miR-9-5p expression is significantly higher in SCCs than in ACs (Figure 1a) [8]. MiR-9-5p expression was higher in SCCs and ACs harboring a copy number gain of *miR-9-1* compared to carcinomas without a 1q gain (Figure 1b). To examine whether low expression levels of miR-9-5p in ACs could also be explained by hypermethylation of one or more of the three miR-9-5p precursor genes, the methylation status of the three miR-9 promoter regions were analyzed by qMSP in 24 SCC and 17 AC tissue samples (Figure 1c). Median methylation levels of all three precursors were elevated in ACs compared to SCCs. For *miR-9-1*, this difference in promoter methylation was significant. Further stratification of tissue samples by hrHPV type (HPV16 vs. other hrHPV types) revealed that the difference in *miR-9-1* and *miR-9-2* methylation between SCCs and ACs is particularly large in HPV16-positive carcinomas (Figure 1d). Stratification by other hrHPV types was not possible due to low sample numbers.

Consistent with this, expression of miR-9-5p was found to be significantly higher in HPV16-positive SCCs (*n* = 145) than in HPV16-positive ACs (*n* = 27) in whole miRNome sequencing data obtained from the TCGA depository (Table S2) [32]. No differences in miR-9-5p expression were found between HPV18-positive SCCs (*n* = 28) and ACs (*n* = 11). In line with our methylation data, moreover, there was no difference in miR-9-5p expression between HPV16- (*n* = 145) and HPV18-positive SCCs (*n* = 28), while miR-9-5p was significantly higher expressed in HPV18-positive ACs (*n* = 11) than in ACs associated with HPV16 (*n* = 27).

### 3.2. miR-9-5p Expression Is Increased in HPV16-Positive But Not in HPV18-Positive Cells

**Figure 2:**
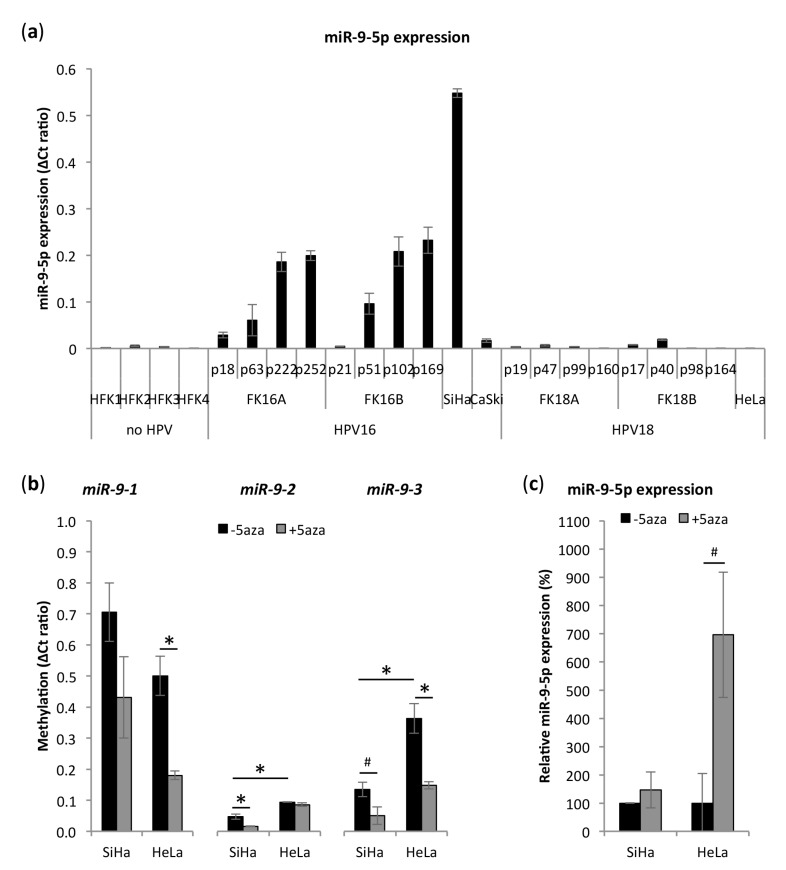
miR-9-5p expression and methylation in cells representing different stages of cervical carcinogenesis. (**a**) Expression of miR-9-5p in primary keratinocytes (HFK), HPV16- and HPV18-immortalized keratinocytes (FK16A/B and FK18A/B) at different passages, and cervical cancer cell lines SiHa, CaSki, and HeLa; (**b**) methylation of miR-9 precursors in SiHa and HeLa cells upon treatment demethylation agent 5-aza-2’-deoxycytidine (5aza) (**c**) and the resulting relative expression of mature miR-9-5p expression after 5aza treatment. # *p* < 0.1, * *p* < 0.05.

In accordance with our tissue data, the expression level of mature miR-9-5p was significantly higher in SiHa (SCC, HPV16) cervical cancer cells than in primary keratinocytes (HFK), HeLa (AC, HPV18), and CaSki (SCC, HPV16) cervical cancer cells (Figure 2a). CaSki is a cervical cancer cell line derived from a SCC metastasis in the small intestine and exhibited intermediated miR-9-5p expression levels. No differences between HeLa and HFKs were observed. To further investigate the relation of miR-9-5p expression and hrHPV type, we also included four HPV16- and HPV18-transformed keratinocyte cell lines. These cell lines originate from the same primary cells and have been shown to mimic hrHPV-induced transformation in vitro upon prolonged culturing [20,24,33,34,35]. This offers the possibility to investigate molecular changes in a time-dependent manner and to study HPV16- and HPV18-specific alterations within the same genetic background. Here, analysis of consecutive passages of hrHPV-transformed keratinocyte cell lines showed increasing miR-9-5p expression in HPV16-transformed keratinocytes FK16A and FK16B. HPV18-transformed keratinocytes FK18A and FK18B exhibited lower endogenous levels of miR-9-5p, mostly comparable to those in HFKs and HeLa. As high passages (≥ 160) of hrHPV-transformed keratinocytes display the highest degree of transformation, they can be best compared to cervical cancer cell lines. High expression of miR-9-5p in high passages of FK16A and FK16B and low expression in high passages of FK18A and FK18B are therefore in line with results obtained in HPV16-positive SiHa and HPV18-positive HeLa cells, respectively.

### 3.3. miR-9-5p Expression Is Regulated by DNA Methylation

To determine whether miR-9-5p expression in cervical cancer cells is methylation-dependent, SiHa and HeLa cells were treated with demethylation agent 5-aza-2’-deoxycytidine (5aza). Baseline methylation of *miR-9-1* was higher in SiHa, while *miR-9-2* and *miR-9-3* had higher methylation levels in HeLa (Figure 2b). 5aza treatment greatly reduced methylation of *mir-9-1* and *mir-9-3* in both SiHa and HeLa cells. *Mir-9-2* methylation was largely reduced in SiHa, but not in HeLa. Expression of mature miR-9-5p increased 1.5-fold in SiHa and 7.0-fold in HeLa treated with 5aza (Figure 2c), respectively, indicating that DNA methylation is more relevant for the regulation miR-9-5p expression in HeLa than in SiHa. All three precursors were not detectably methylated in HFKs (data not shown).

### 3.4. miR-9-5p Plays Opposing Functional Roles Cervical Cancer Cells

**Figure 3:**
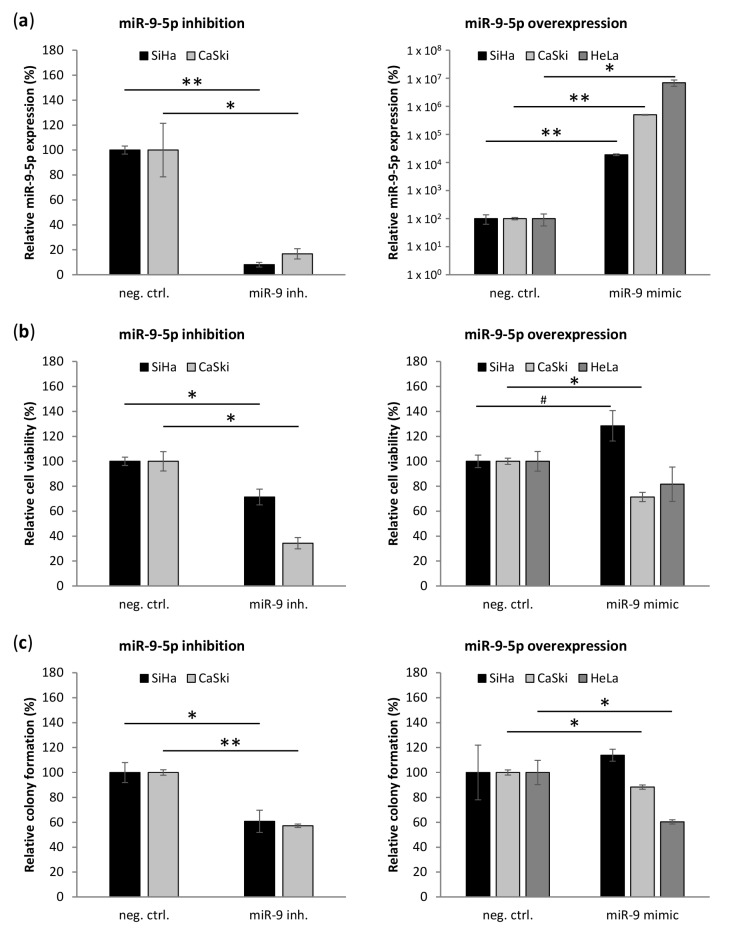
Effect of miR-9-5p on cervical cancer cell lines. (**a**) miR-9-5p levels; (**b**) cell viability, and (**c**) colony formation in soft-agar upon inhibition (left panel) and overexpression (right panel) of miR-9-5p in cervical cancer cell lines SiHa, CaSki, and HeLa relative to the respective negative control. The miR-9-5p inhibition experiment was not performed in HeLa due to very low endogenous levels of miR-9-5p in this cell line. # *p* < 0.1, * *p* < 0.05, ** *p* < 0.005.

Differential expression of miR-9-5p in cervical SCC and AC suggests that miR-9-5p plays a different role in the two histotypes. We therefore investigated the effect of miR-9-5p inhibition in SiHa and CaSki and overexpression in SiHa, CaSki, and HeLa (Figure 3). Inhibition was not tested in HeLa cells because of already low endogenous miR-9-5p levels. Inhibition of miR-9-5p largely reduced miR-9-5p levels in SiHa and CaSki (Figure 3a). The effect of miR-9-5p mimics on miR-9-5p levels seemed to be inversely correlated to endogenous miR-9-5p levels, SiHa exhibiting the lowest and HeLa the highest increase in miR-9-5p levels. Consistent with an oncogenic role in SCC, miR-9-5p inhibition reduced cell viability in SiHa, while cell viability was slightly increased upon overexpression of miR-9-5p (Figure 3b). A reduction of cell viability upon miR-9-5p inhibition was even more pronounced in CaSki. Interestingly, cell viability of CaSki was also decreased upon miR-9-5p overexpression, indicating a dose-dependent effect of miR-9-5p in CaSki. Overexpression of miR-9-5p reduced cell viability in HeLa to a minor extent. Comparable to the effect on cell viability, anchorage-independent growth (colony formation in soft-agar) was reduced upon miR-9-5p inhibition in SiHa and CaSki (Figure 3c). While overexpression of miR-9-5p slightly increased colony formation of SiHa, anchorage-independent growth was slightly reduced in CaSki. Colony formation was largely decreased in HeLa upon overexpression of miR-9-5p.

### 3.5. Transcription Factor TWIST1 Is a Direct Target of miR-9-5p

**Figure 4:**
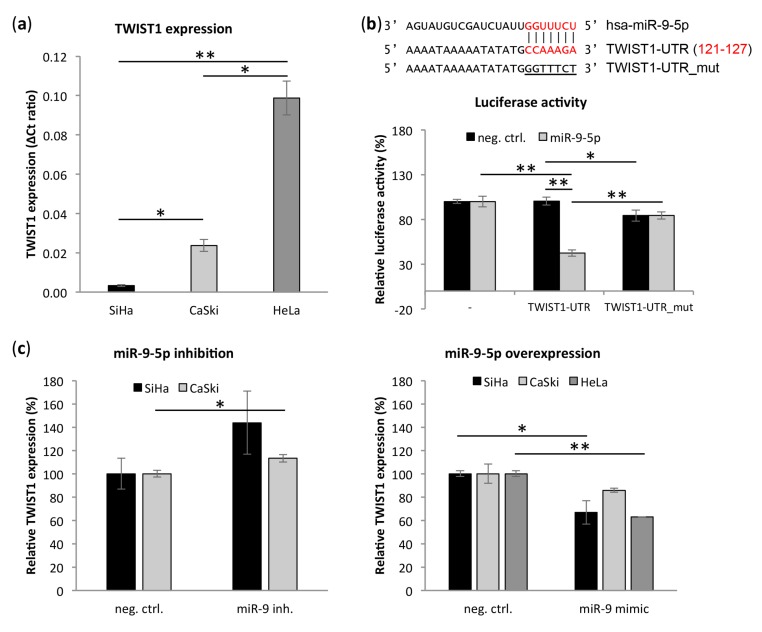
miR-9-5p directly targets transcription factor twist family bHLH transcription factor 1 (TWIST1). (**a**) Endogenous expression of TWIST1 mRNA in cervical cancer cell lines SiHa, CaSki, and HeLa; (**b**) binding site of miR-9-5p in the TWIST1-3UTR with the seed sequence indicated in red. Dual-luciferase reporter assay in HEK293 cells transiently transfected with a negative control or miR-9-5p mimics in combination with either an empty pmiRGLO vector (-), a pmiRGLO-TWIST1-UTR construct (TWIST1_UTR) or a pmiRGLO-TWIST1_UTR construct with mutated binding site of miR-9-5p (TWIST1-UTR_mut); (**c**) TWIST1 mRNA expression upon inhibition and overexpression of miR-9-5p relative to the respective negative control of TWIST1 mRNA in cervical cancer cell lines SiHa, CaSki, and HeLa. * *p* < 0.05, ** *p* < 0.005.

Cancerous epithelial cells achieve anchorage independence by induction of epithelial-to-mesenchymal transition (EMT), thereby overcoming detachment-induced cell death (anoikis) [36]. MiR-9-5p has previously been implicated in this process by targeting CDH1 [12,16,37,38,39,40,41]. Interestingly, using TargetScan v7 [42] and RNA22 v2.0 [43] algorithms we identified transcription factor and EMT regulator TWIST1 as a novel potential miR-9-5p target. In our cervical cancer cell lines SiHa, CaSki, and HeLa, low miR-9-5p expression coincided with high TWIST1 expression and vice versa (Figure 2a and Figure 4a). TWIST1 had the highest endogenous expression in HeLa, five times higher than in CaSki and 30 times higher than in SiHa. Moreover, TWIST1 expression was borderline significantly negatively correlated to miR-9-5p expression in SCCs (*n* = 161) from the TCGA network (Table S3) [32]. In the same dataset, TWIST1 and miR-9-5p expression were significantly positively correlated in ACs (*n* = 39). To study a potential direct interaction between miR-9-5p and the TWIST1 3’UTR, a luciferase reporter assay was performed using the predicted binding site of miR-9-5p in the TWIST1 3’UTR (Figure 4b) [42,43]. Co-transfection of the TWIST1-UTR reporter and miR-9-5p in HEK293 cells decreased luciferase activity compared to either co-transfection of the reporter with a non-targeting control sequence (negative control) or the empty luciferase vector with miR-9-5p (Figure 4b). Directed mutagenesis of the predicted miR-9-5p seed sequence (TWIST1-UTR_mut) abolished the reduction in luciferase activity observed with the wild type vector, confirming a direct interaction between miR-9-5p and the TWIST1 3’UTR. In support of a miR-9-5p mediated regulation of TWIST1, TWIST1 expression was highly increased upon inhibition of miR-9-5p in SiHa with high endogenous miR-9-5p expression (Figure 4c). In CaSki, having intermediate endogenous miR-9-5p levels, miR-9-5p inhibition slightly increased TWIST1 expression. Overexpression of miR-9-5p in HeLa with low endogenous miR-9-5p, on the other hand, led to a decrease in TWIST1 mRNA. TWIST1 levels were also reduced upon miR-9-5p overexpression in SiHa and CaSki.

### 3.6. miR-9-5p Regulates Expression of CDH1 and CDH2

**Figure 5:**
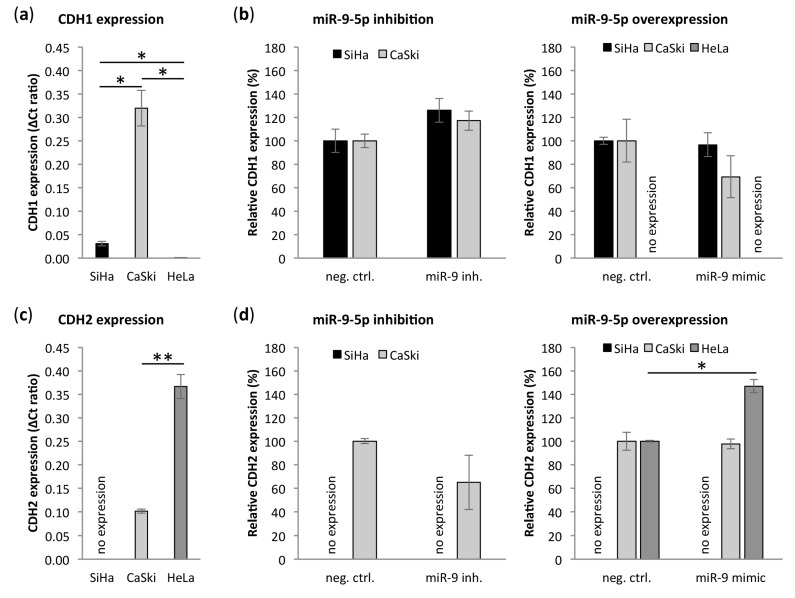
Effect of miR-9-5p on E-cadherin (CDH1) and N-cadherin (CDH2). Endogenous expression of (**a**) CDH1 and (**c**) CDH2 mRNA in cervical cancer cell lines SiHa, CaSki and HeLa. Expression of (**b**) CDH1 and (**d**) CDH2 upon inhibition and overexpression of miR-9-5p relative to the respective negative control. CDH1 expression remained undetected in HeLa after transfection and CDH2 expression remained undetected in SiHa. * *p* < 0.05, ** *p* < 0.005.

In addition to translational repression of CDH1 by miR-9-5p, TWIST1 has previously been described to inhibit CDH1 on a transcriptional level [44,45,46,47]. In addition, TWIST1 transcriptionally activates the mesenchymal protein N-cadherin (CDH2) [45,46,47]. To investigate whether the direct interaction between miR-9-5p and TWIST1 can (partly) explain the different role of miR-9-5p in cervical SCC and AC, we analyzed expression of CDH1 and CDH2 in cervical cancer cells. Endogenous CDH1 expression was very high in CaSki, while it was just above the detection limit in HeLa (Figure 5a). In SiHa, CDH1 levels were 10 times lower than in CaSki, yet clearly higher than in HeLa. Upon miR-9-5p inhibition, CDH1 increased in SiHa and CaSki (Figure 5b). Overexpression of miR-9-5p did not affect CDH1 levels in SiHa, possibly due to already high levels of miR-9-5p in this cell line. In CaSki, CDH1 levels were reduced upon miR-9-5p overexpression. CDH1 expression remained undetectable in transfected HeLa cells. Comparable to TWIST1, CDH2 had high endogenous levels in HeLa, intermediate levels in CaSki, and remained undetected in SiHa (Figure 5c). CDH2 expression decreased upon miR-9-5p inhibition in CaSki and increased upon miR-9-5p overexpression in HeLa (Figure 5d). Comparable to TWIST1, CDH2 expression was significantly negatively correlated to miR-9-5p expression in SCCs and borderline significantly positively correlated to miR-9-5p expression in ACs from the TCGA data (Table S3) [32].

## 4. Discussion

MiR-9-5p has been reported to play opposite roles in different cancer types by either promoting or suppressing malignant transformation [17]. In cervical cancer, differential expression of miR-9-5p in SCC and AC even suggests a dual function of miR-9-5p between the two histotypes. Our present data indicate that the differential deregulation of miR-9-5p depends on both the underlying cell type and the hrHPV type present. Moreover, we demonstrate that miR-9-5p functions as oncomiR in SCC-derived cells, while it exerts a tumor suppressive role in AC-derived cells. In addition to the previously established interaction between miR-9-5p and CDH1, the identification of transcription factor TWIST1 as a novel miR-9-5p target further implicates miR-9-5p in epithelial-to-mesenchymal transition (EMT).

As described above, increased expression of miR-9-5p in cervical SCC is partly attributable to a copy number gain of the *mir-9-1* gene located on chromosome 1q [8]. This chromosomal gain is found in 29–78% of SCC and 22–57% of AC, which might partly explain the difference in miR-9-5p expression between the two histotypes [20,21]. In accordance with previous data, we find higher methylation levels of all miR-9-5p precursors in AC compared to SCC [22], even though this difference was only significant for *miR-9-1* in our data. Methylation of the *miR-9-1* promoter in ACs harboring a 1q gain might be necessary to compensate for the increased miR-9-5p gene dosage in this histotype. Unfortunately, the copy number data was only available for a small number of tissue samples included in our study. The relation between 1q gain, *miR-9-1* methylation, and miR-9-5p expression therefore warrants further investigation.

MiR-9-5p was also found to be upregulated in hrHPV-positive oral and oropharyngeal head and neck cancers [48,49]. The large proportion of HPV16-positive carcinomas included in these studies suggest a link between high miR-9-5p expression and HPV16 in SCC. In line with this, miR-9-5p expression is increased in HPV16 E6/E7 expressing keratinocytes and HPV16 E6 was shown to upregulate miR-9-5p expression in a p53-independent manner [50,51]. Stratification of our methylation data and TCGA miR-9-5p expression data by hrHPV type further indicates that miR-9-5p expression in cervical cancer is hrHPV type-dependent. Similarly, Nilsen et al. found that miR-9-5p expression is significantly higher in HPV16-positive than in HPV18-positive cervical SCCs and Liu et al. reported higher levels of miR-9-5p in HPV16-positive than in HPV18-associated cervical tumors without further specification of tumor histology [51,52]. Abundance of miR-9-5p in HPV16-transformed keratinocytes and SiHa (HPV16), as well as low levels as miR-9-5p in HPV18-transformed keratinocytes and HeLa (HPV18) in the present study further support the notion that miR-9-5p expression depends on hrHPV type. Considering both methylation and expression data it may be suggested that the histotype effects are more pronounced than the hrHPV type effects.

Differential expression between cervical cancer histotypes suggests a dual role of miR-9-5p in cervical cancer, as has previously been observed between different human cancers [17]. In fact, we demonstrate that miR-9-5p promotes cell viability and anchorage-independent growth of cervical SCC cell line SiHa, whereas the opposite effect is observed in HeLa (AC). Inhibition of miR-9-5p had a smaller effect on cell viability in SiHa than in CaSki (metastasis, HPV16), probably due to the high endogenous levels of miR-9-5p SiHa. Previous studies have reported similar effects of miR-9-5p in SiHa and HeLa [18,22]. While FOXO3 and IL6 were shown to be targeted by miR-9-5p in these studies, we could not confirm downregulation of FOXO3 and IL6 upon miR-9-5p overexpression (Figure S1).

Our results on anchorage-independent growth, as well as the described effects of miR-9-5p on migration implicate miR-9-5p in EMT. EMT describes the loss of epithelial characteristics and the acquisition of a mesenchymal phenotype [53]. This process has been closely linked to invasiveness and metastasis of cancer cells [54]. In cervical cancer, EMT promotes malignant progression and metastasis, as well as chemo- and radio-resistance [55]. Here, we identified transcription factor and mesenchymal marker TWIST1 as a novel direct target of miR-9-5p. TWIST1 has previously been shown to promote EMT and a cancer stem-like cell (CSLC) phenotype by transcriptional activation of mesenchymal markers and CSLC-associated proteins in HeLa [45,56]. In the same cell line, inhibition of TWIST1 reduced proliferation, which is concordant with the reduction of cell viability observed upon overexpression of miR-9-5p in HeLa in our study [57]. TWIST1 mediates EMT by transcriptional suppression of epithelial protein CDH1 and transcriptional activation of mesenchymal marker CDH2 [44,45,46,47]. Interestingly, CDH1 has also been established as a direct target of miR-9-5p in a multitude of human cancers [12,16,37,38,39,40,41]. In cervical cancer, downregulation of CDH1 has been observed [58,59,60]. Together with our miR-9-5p inhibition data in SiHa and CaSki, this indicates that miR-9-5p also targets CDH1 in cervical SCC, even though the effect of miR-9-5p on TWIST1 was more pronounced. In HeLa, where miR-9-5p expression is low, low and undetectable levels of CDH1 might therefore be mediated by high levels of TWIST1 rather than miR-9-5p [59]. In support of that and similar to our findings, Zhang et al. observed no effect of miR-9-5p on CDH1 expression in HeLa [22]. High CDH2 expression in cervical cancer cells coincided with low levels of miR-9-5p and high levels of TWIST1, suggesting a potential indirect effect of miR-9-5p on CDH2 via TWIST1. In line with this, CDH2 expression is significantly higher in ACs than in SCCs in publicly available expression data from the TCGA network (data not shown) [32].

**Figure 6:**
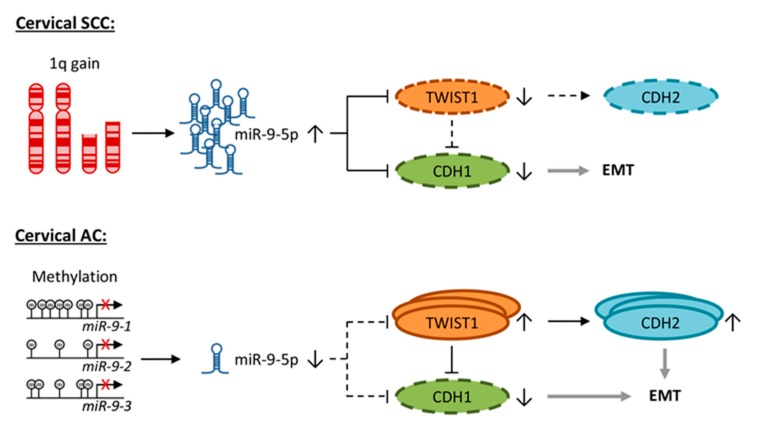
Concept of the effect of miR-9-5p on EMT in cervical cancer. TWIST1 and CDH1 are direct targets of miR-9-5p. TWIST1 inhibits transcription of CDH1 and promotes CDH2 expression. In cervical SCC, chromosomal gain of 1q causes miR-9-5p overexpression. Here, miR-9-5p inhibits translation of TWIST1 and CDH1 and low expression of CDH1 promotes epithelial-to-mesenchymal transition (EMT). In cervical AC, miR-9-5p expression is low due to promoter methylation of its precursor genes, particularly *miR-9-1*. TWIST1 is not inhibited by miR-9-5p and mediates suppression of CDH1, as well as upregulation of CDH2, thereby promoting EMT.

Based on our findings, we propose two different mechanisms for the involvement of miR-9-5p in the induction of EMT in cervical cancer (Figure 6): In SCC, abundance of miR-9-5p caused by a chromosomal gain of 1q leads to degradation of both TWIST1 and CDH1 [8]. CDH2 expression is low in the absence of TWIST1. In AC, miR-9-5p expression is repressed by methylation of its precursors. As a result, TWIST1 is not suppressed, high levels of TWIST1 inhibit transcription of CDH1 and activate transcription of CDH2. In cervical SCC, the downregulation of CDH1 by miR-9-5p might be sufficient to mediate an EMT phenotype, while both the TWIST1-mediated inhibition of CDH1 and upregulation of CDH2 might be necessary to maintain a malignant phenotype in cervical AC. However, the here presented data provides stronger support for the proposed mechanism in cervical SCC than AC. Our suggested concept therefore warrants further investigation.

Taken together, our results suggest that miR-9-5p expression depends on both cervical cancer histotype and hrHPV type. Identification of TWIST1 as a novel miR-9-5p target further implicated miR-9-5p in EMT. In SCC, miR-9-5p acts as oncogene by targeting TWIST1, as well as by directly and indirectly targeting CDH1. In AC, miR-9-5p exerts a tumor suppressive role and the EMT phenotype is achieved by low levels of miR-9-5p, which enable the upregulation of CDH2 via TWIST1.

## Supplementary Materials

The following are available online at https://www.mdpi.com/2073-4409/9/1/65/s1. Table S1: Primer sequences; Table S2: Expression of miR-9-5p in whole miRNome sequencing data from the TCGA depository [32]; Table S3: Correlation of miR-9-5p expression with expression of TWIST1, CDH1, and CDH2; Figure S1: Effect of miR-9-5p on IL6 and FOXO3 in cervical cancer cell lines.
