# Supplementary material for: miR-9-5p Exerts a Dual Role in Cervical Cancer and Targets Transcription Factor TWIST1
Source: Cells. 2019 Dec 26;9(1):65. doi: 10.3390/cells9010065 (PMC7017350; doi:10.3390/cells9010065)
Supplement: Supplementary file 1 [file cells-09-00065-s001.pdf]

Table S1. Primer sequences.

(**a**) **miR-9 quantitative MSP**

Gene	Primers (5′-3′)	Size (bp)
miR-9-1	F: GAAATGTCGTTCGAATTAGTGAG	123
	R: GAAAAAAACCGACCTCACGA	
	P: GGTGTTGGTTAGTTTTCGCGGTTTTAGTTTTTC -6FAM	
miR-9-2	F: CGTTGTCGGAGATTATTTGTTG	177
	R: ATACCGAAAAATAATCGTCCA	
	P: ATTTTGAAGGTAATAGATTTCGTTTGGATGTTTTAGTCGC -YY	
miR-9-3	F: TGTGCGTGTGTTTGTTTATTT	135
	R: CTTAACCAATACCGCTACCG	
	P: TTCGGGTACGGCGTTCGTTTAGGTTTTCG -DFO	
ACTB	F: TGGTGATGGAGGAGGTTTAGTAAGT	133
	R: AACCAATAAAACCTACTCCTCCCTTAA	
	P: ACCACCACCCAACACACAATAACAAACACA -CY5	

bp, base pairs; F, Forward primer; R, Reverse primer; P, probe

(**b**) **TaqMan quantitative RT-PCR**

Gene	Primers (5′-3′)	Size (bp)
CDH1	F: TTGACGCCGAGAGCTACAC	93
	R: GACCGGTGCAATCTTCAAA	
	P: GCGTCCTGGGCAGAGT -6FAM	
CDH2	F: CCACCTTAAAATCTGCAGGC	100
	R: GTGCATGAAGGACAGCCTCT	
	P: GGAAAAAGAAAAGTACAATATGAGAGCAGT -6FAM	
snRNP U1A	F: TCCTCACCAACCTGCCAGA	72
	R: TGAAGCCAGGGAACTGATTGA	
	P: AGACCAACGAGCTCATGCTGTCCATG -6FAM	
TWIST1	F: GTCCGCGTCCCACTAGC	90
	R: TCCATTTTCTCCTTCTCTGGAA	
	P: GCAGGGCCGGAGACCTA -6FAM	

bp, base pairs; F, Forward primer; R, Reverse primer; P, probe

(**c**) **TWIST 3′UTR cloning & mutagenesis**

Gene	Primers (5′-3′)
3'UTR-TWIST1	F: TCGAGAGCTCTA GCCGGAGACCTAGATGTCATTGTTTC
	R: ACTGCTCGAGTA GCCCGTCTGGGAATCACTGTC
3'UTR-TWIST1_mut	F: tctTTTTCTTGGAAATTAGAAGAGC
	R: aaccCATATATTTTTATTTTTAGTTATCCAG

bp, base pairs; F, Forward primer; R, Reverse primer; restriction site (F: SacI, R: XhoI); lower case: induced mutation

Table S2. Expression of miR-9-5p in whole miRNome sequencing data from the TCGA depository [32]. Data of 211 cervical carcinomas, which were either positive for HPV16 or HPV18, were included. * $p < 0.05$.

Comparison	p-value	Direction
by HPV type:		
HPV16: SCC (n=145) vs AC (n=27)	0.001 *	higher in SCC
HPV18: SCC (n=28) vs AC (n=11)	0.386	higher in SCC
by histotype:		
SCC: HPV16 (n=145) vs HPV18 (n=28)	0.209	higher in HPV18
AC: HPV16 (n=27) vs HPV18 (n=11)	0.021 *	higher in HPV18

Table S3. Correlation of miR-9-5p expression with expression of TWIST1, CDH1, and CDH2 per histotype. 200 cervical carcinomas were included, for which both whole miRNome and whole transcriptome sequencing data from the TCGA depository was available [32]. Spearman correlation coefficient rho and the associated p-value were assessed. # $p < 0.1$, * $p < 0.05$.

Spearman correlation	TWIST1		CDH1		CDH2	
	Rho	p-value	Rho	p-value	Rho	p-value
SCC (n=161)	-0.138	0.082 #	0.031	0.696	-0.167	0.035 *
AC (n=39)	0.367	0.022 *	-0.117	0.478	0.281	0.083 #

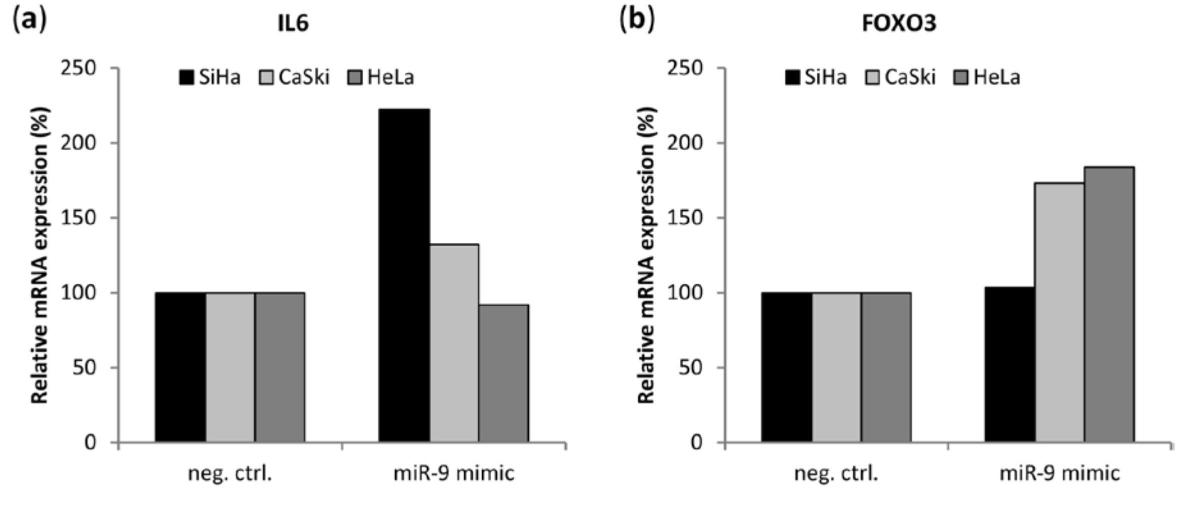

Figure S1. Effect of miR-9-5p on IL6 and FOXO3 in cervical cancer cell lines. Expression of (**a**) IL6 and (**b**) FOXO3 mRNAs upon overexpression of miR-9-5p in cervical cancer cell lines SiHa, CaSki, and HeLa relative to the respective negative control. FOXO3 melting curve analysis indicated aspecific products and FOXO3 expression data should therefore be interpreted with caution.
